# Physical activity and weight decline before frailty: Implications for frailty prevention and reverse causation

**DOI:** 10.1093/geroni/igaf122.2375

**Published:** 2025-12-31

**Authors:** Hee-kyoung Nam, Jie Shen, Andrea Wershof Schwartz, Sung-il Cho, Changzheng Yuan, Yuan Ma

**Affiliations:** Seoul National University College of Medicine, Boston, Massachusetts, United States; Harvard T.H. Chan School of Public Health, Boston, Massachusetts, United States; Harvard T.H. Chan School of Public Health, Boston, Massachusetts, United States; Seoul National University, Seoul, Republic of Korea; Zhejiang University, Hangzhou, Zhejiang, China (People’s Republic); Harvard T.H. Chan School of Public Health, Boston, Massachusetts, United States

## Abstract

Background Physical inactivity and mid-life obesity are modifiable risk factors for frailty. However, declines in physical activity and weight loss may co-occur in the years preceding the onset of frailty. It is undetermined whether monitoring concurrent changes in physical activity and body weight can improve the early detection of frailty and to what extent their relationship is influenced by reverse causation. Methods We examined the longitudinal changes in physical activity and body weight over 8 years and the incidence of frailty over a median follow-up of 8 years among 5,759 community-dwelling older adults from the nationally representative Health and Retirement Study (US). We further assessed physical activity and BMI measured at different years (0, 2, 4, 6, and 8 years, respectively) preceding frailty assessment. Findings A total of 1293 incident frailty cases were documented. Decreased physical activity (adjusted hazard ratio [HR]:1.22; 95% CI, 1.06-1.39) and weight loss (HR = 1.20; 95% CI, 1.05-1.37) were independently associated with increased frailty risk after adjusting for initial physical activity and BMI and other covariates, with the highest risk observed in individuals experiencing both. The association between lower physical activity and increased risk of frailty appeared more pronounced when physical activity was assessed closer to frailty diagnosis, whereas the association between obesity and frailty was attenuated. Discussion Tracking changes in physical activity and body weight may be an easy and effective approach to help identify high-risk individuals for timely frailty intervention. Reverse causation may impact association estimates and causal interpretations even in non-frail older adults.

